# ﻿Numerical analyses of seed morphology and its taxonomic significance in the genus *Oxytropis* DC. (Fabaceae) from northwestern China

**DOI:** 10.3897/phytokeys.222.96990

**Published:** 2023-03-21

**Authors:** Xiang Zhao, Yingying Liu, Jigang Li, Hui Zhang, Lingyun Jia, Qinzheng Hou, Kun Sun

**Affiliations:** 1 College of Life Sciences, Northwest Normal University, Lanzhou 730070, Gansu, China Northwest Normal University Lanzhou China

**Keywords:** China, cluster analysis, *Oxytropis*, PCA, seed morphology, SEM, taxonomy

## Abstract

The lack of diagnostic taxonomic characteristics in some species complexes leave the species delimitation of *Oxytropis* DC. unresolved. Seed morphological features have proved to be useful diagnostic and taxonomic characteristics in Fabaceae. However, there are few systematic studies on the seed characteristics of *Oxytropis*. Here, we used scanning electron and stereoscopic microscopy to investigate the seed characteristics of 35 samples obtained from 21 *Oxytropis* species from northwest China. Our examination showed two main types of hilum positions, terminal and central, and five different types of seed shapes: prolonged semielliptic, reniform, prolonged reniform, quadratic, and cardiform. Seven different sculpturing patterns were identified: scaled, regulated, lophate with stellated testa cells, simple reticulate, rough, compound reticulate, and lophate with rounded testa cells. The seeds ranged from 1.27 to 2.57 mm in length and from 1.18 to 2.02 mm in width, and the length-to-width ratio ranged from 0.89 to 1.55 mm. The seed shape was constant within species and was useful for species delimitation within the genus *Oxytropis* when combined with other macroscopic traits. In contrast, the sculpturing patterns were highly variable at the species level and could not be used for species identification. Results of the cluster analysis and principal component analysis (PCA) indicated that the seed traits of *Oxytropis* species are useful for taxa identification at the species level, but have low taxonomic value at the section level.

## ﻿Introduction

The genus *Oxytropis* DC. belongs to the tribe Galegeae (Fabaceae: Papilionoideae). It has been reported to be one of the largest groups of angiosperms, comprising approximately 330 species. The genus is distributed mainly in the cold mountainous regions of Asia, Europe, and North America (Polhill 1981; Zhu et al. 2010). It is thought to have been derived from *Astragalus* L. approximately 12–16 Ma, with which it shares many morphological features (Wojciechowski 2005). The genus *Oxytropis* is distinguished from *Astragalus* by beaked keels, asymmetrical leaflets, and acaulescent habit (Barneby 1952). Likely because of its relatively recent diversification, many taxonomic relationships within *Oxytropis* remain problematic (reviewed in Welsh 2001).

The genus *Oxytropis* was first established in 1802 by De Candolle (De Candolle 1802). It included 33 species, and he divided them into three groups according to whether stipules are adherent to stems or not and whether leaflets are opposite, verticillate, or neither. Bunge’s (1874) comprehensive treatment of *Oxytropis* species in Eurasia identified four subgenera, 19 sections, and 181 species. His research also marked the beginning of modern *Oxytropis* research. Vassilczenko (1948) revised the work on *Oxytropis* in the Flora of USSR and separated the genus into six subgenera, 21 sections, and 276 species. Pavlov (1961) divided *Oxytropis* into four subgenera, 15 sections, and 124 species in the Flora of Kazakhstan. Leins and Merxmüller (1968) compiled 24 species, three subspecies, one variety, and two suspected species from Europe and divided them into two groups. Zhang (1998) recognized six subgenera, 146 species, 12 varieties, and three forms in Flora Reipublicae Popularis Sinicae. In contrast, Zhu and Ohashi (2000) recognized 125 species and four varieties in China. Welsh (2001) revised the genus *Oxytropis* in North America to include 57 taxa in only 22 species. Later, Zhu et al. (2010) taxonomically revised the genus in China and reported that it comprises three subgenera and 20 sections containing 133 species. These previous treatments of *Oxytropis* clarified many taxonomic problems. However, the lack of diagnostic taxonomic characteristics in some *Oxytropis* complexes has led to difficulties and differences in species delimitation, leaving the internal classification of *Oxytropis* unresolved.

Seed morphological features, such as seed shape, hilum shape, sculpturing pattern, and size, have been proven to be useful diagnostic and taxonomic characteristics in some genera of Fabaceae and other families (Lersten and Gunn 1981; Solum and Lockerman 1991; López et al. 2000; Al-Gohary and Mohamed 2007; Salimpour et al. 2007; Vural et al. 2008; Venora et al. 2009; Zorić et al. 2010; Celep et al. 2012; Kaya and Dirmenci 2012; Lantieri et al. 2013; Kamala and Aydin 2018; Rashid et al. 2018; Shemetova et al. 2018; Rashid et al. 2020). In the genus *Trifolium* L., Salimpour et al. (2007) reported that seed characteristics such as sculpturing pattern, shape, size, and hilum position, can be used as taxonomic markers within the section Lotoidea. In contrast, Zorić et al. (2010) concluded that the seed characteristics do not support infrageneric classification of *Trifolium*. Similarly, Shemetova et al. (2018) reported that seed shapes, colours, sizes, surface sculptures, and hilum positions are very diverse in *Astragalus*, and they emphasized that the systematic importance of seed characteristics needs to be evaluated in a phylogenetic context. However, Vural et al. (2008) found that *Astragalus* seed sculpturing pattern and seed shape can be used as taxonomically significant characteristics at the species level, if supported by other macromorphological characteristics. López et al. (2000) found that seed colour, weight, shape, and size, presence of an aril, and hilum position can be used as diagnostic characteristics for segregating two subtribes and delimiting lower taxonomic levels in the tribe Genisteae. Similarly, Rashid et al. (2020) concluded that seed shape, sculpturing pattern, and size are valuable characteristics for the identification and delimitation of species in the tribes Astragaleae and Trifolieae. Kamala and Aydin (2018) and Rashid et al. (2018) also reported that seed characteristics (coat, shape, colour, seed size, etc.) can be used to identify taxa in the tribe Vicieae.

Seeds of *Oxytropis* species were first studied by Solum and Lockerman (1991), who documented the seed coat patterns of *Oxytropisriparia* Litv. and *Oxytropiscampestris* (L.) DC. Bojňanský and Fargašová (2007) studied the seeds of four European *Oxytropis* species and recorded their size, colour, and other information. Farrington et al. (2008) studied the morphological properties of seeds of 15 Alaskan *Oxytropis* taxa and found that seed coat micromorphology and anatomy can distinguish it from the genus *Astragalus*. Meyers et al. (2013) analysed the seed characteristics of 22 *Oxytropis* species in Alaska and concluded that seed coat types are highly variable at the species level and cannot be used for species identification. Erkul et al. (2015) studied the morphological properties of seeds of 13 Turkish *Oxytropis* taxa and found that seed characteristics have low taxonomic value in distinguishing subgenera, sections, and species. The infraspecific variation in seed traits has not been well addressed in most of the abovementioned studies because of sampling limitations. Only Meyers et al. (2013) studied whether seed traits were stable within species, along with studying the correlation between seed traits and the environment; however, they did not conduct any systematic analyses, such as cluster analysis.

Numerical taxonomy, also known as phenetics, mathematical taxonomy and multivariate morphometrics (Singh 2019), is mainly based on the overall affinity (similarity) at any taxonomic level. Quantitative traits have long been overlooked in taxonomic studies until numerical methodologies, such as cluster analysis, started to be widely applied in species delimitation (Thien et al. 1975). Recently, dendrograms and cladograms have been used instead of subjective analyses in many studies on the seed morphology of Fabaceae (Erkul et al. 2015; Fayed et al. 2019; Abusaief and Boasoul 2021). However, quantitative seed traits of the genus *Oxytropis*, such as length, width, length/width ratio, and weight, have not received much attention in taxonomic studies, possibly because these traits are considered fluctuating, and this fluctuation is random or excessive.

Northwest China is one of the main distribution regions of the genus *Oxytropis* (Zhang 1998; Zhu et al. 2010), but there is little research on the seed characteristics of *Oxytropis* in this area. Here, we carried out the first numerical analysis and microscopic investigation of 35 samples belonging to 21 *Oxytropis* species from northwest China using scanning electron and stereoscopic microscopy to elucidate the taxonomic significance of their seed micromorphology.

## ﻿Materials and methods

The present study was mainly based on seeds collected in the field, with only a few seeds obtained from herbarium vouchers housed at the herbarium of Northwest Normal University (HWTC; Table 1). Voucher specimens collected from wild seeds are also kept at HWTC. The investigated species and their sources are listed in Table 1, and the classification of genera by Zhu et al. (2010) was adopted. Seed morphology was examined using a stereoscopic microscope (Leica M205 FA). For measuring seed length and width among the samples from the field, 80 mature and representative seeds per population were measured, while among the samples from herbarium specimens, 30 seeds per specimen were measured. The minimum–maximum range, mean, standard deviations in seed length and width, and length/width ratio were calculated. For SEM, the selected representative material was directly mounted onto aluminium stubs with double adhesive tape and coated with gold prior to observation with a HITA-CHIS-450 scanning electron microscope (NWNU University) at 25 kV.

**Table 1.** List of examined taxa with collection details.

Section	Code	Species	Locality	Coordinates	Voucher	Collection date
Section Xerobia	1	*O.ciliata* Turcz.	Yueliang Mountain	36°25'41.85"N, 105°42'23.71"E	X. Zhao 1947	2019
Section Polyadena	2	*O.muricata* (Pall.) DC.	Maxian Mountain	35°47'46.48"N, 103°58'12.64"E	X. Zhao 1903	2019
	3	*O.muricata* (Pall.) DC.	Tiemu Mountain	35°58'32.21"N, 104°46'31.40"E	X. Zhao 1970	2019
Section Falcicarpae	4	*O.falcata* Bunge	Awangcang wetland park	33°45'32.85"N, 101°41'6.58"E	X. Zhao 1842	2018
	5	*O.falcata* Bunge	Beach of Maqu section of Yellow River	Unknown	Gannan Grassland Team 60B	Unknown
Section Baicalia	6	*O.ochrantha* Turcz.	Xinglong Mountain	35°45'52.41"N, 104°2'21.66"E	X. Zhao 1813	2018
	7	*O.ochrantha* Turcz.	North mountain of Pingliang	35°33'49.11"N, 106°41'2.34"E	X. Zhao 1837	2018
	8	*O.bicolor* Bunge	Unknown	Unknown	Unknown 790043	Unknown
	9	*O.bicolor* Bunge	Tiemu Mountain	35°58'32.21"N, 104°46'31.40"E	X. Zhao 1927	2019
	10	*O.racemosa* Turcz.	Yanchi	37°43'52.02"N, 107°23'55.77"E	X. Zhao 1946	2019
	11	*O.myriophylla* (Pall.) DC.	Erdaogou	35°25'19.39"N, 106°40'6.25"E	X. Zhao 1831	2018
	12	*O.myriophylla* (Pall.) DC.	Anguo	35°38'49.75"N, 106°28'54.92"E	X. Zhao 1833	2018
	13	*O.myriophylla* (Pall.) DC.	Maxian Mountain	35°47'46.48"N, 103°58'12.64"E	X. Zhao 1836	2018
Section Neimonggolicae	14	*O.neimonggolica* C.W.Chang & Y.Z.Zhao	Helan Mountain	38°39'37.76"N, 105°48'34.42"E	X. Zhao 1948	2019
Section Eumorpha	15	*O.imbricata* Kom.	Liancheng National Nature Reserve	36°36'24.65"N, 102°49'34.30"E	X. Zhao 1809	2018
	16	*O.imbricata* Kom.	Taohe River	34°33'28.66"N, 102°34'53.99"E	X. Zhao 1940	2019
	17	*O.coerulea* (Pall.) DC.	Taitong Mountain	35°30'8.94"N, 106°35'54.90"E	X. Zhao 1832	2018
	18	*O.coerulea* (Pall.) DC.	Erdaogou	35°25'19.39"N, 106°40'6.25"E	X. Zhao 1833	2018
	19	*O.holanshanensis* H.C.Fu	Helan Mountain	38°39'37.76"N, 105°48'34.42"E	X. Zhao 1949	2019
Section Mesogaea	20	*O.xinglongshanica* C.W.Chang	Maxian Mountain	35°46'46.16"N, 103°59'19.19"E	X. Zhao 1913	2019
	21	*O.xinglongshanica* C.W.Chang	Xinglong Mountain	35°46'20.53"N, 104°1'2.49"E	X. Zhao 1910	2019
	22	*O.glabra* (Lam.) DC.	Rabah Lake National Nature Reserve	37°42'3.19"N, 107°2'33.46"E	X. Zhao 1950	2019
	23	*O.kansuensis* Bunge	Azi Test Station of LZU	33°39'57.96"N, 101°52'22.44"E	X. Zhao 1819	2018
	24	*O.kansuensis* Bunge	Charlie temple	32°45'7.95"N, 102°3'26.83"E	X. Zhao 1820	2018
	25	*O.taochensis* Kom.	Liupan Mountain	35°33'21.81"N, 106°25'21.54"E	X. Zhao 1838	2018
	26	*O.ochrocephala* Bunge	Nanhuang Mountain	36°22'42.67"N, 105°39'26.20"E	X. Zhao 1952	2019
	27	*O.ochrocephala* Bunge	Xinglong Mountain	35°47'5.17"N, 104°0'0.67"E	X. Zhao 1828	2018
	28	*O.ochrocephala* Bunge	Maxian Mountain	35°46'46.60"N, 103°59'19.33"E	X. Zhao 1953	2019
	29	*O.ochrocephala* Bunge	Jinqiang River	37°13'36.45"N, 102°41'3.46"E	X. Zhao 1840	2018
	30	*O.ochrocephala* Bunge	Hougou Village	35°48'47.34"N, 103°57'53.83"E	X. Zhao 1954	2019
	31	*O.qinghaiensis* Y.H.Wu	Labrang Monastery	35°11'8.91"N, 102°30'37.00"E	X. Zhao 1822	2018
Section Oxytropis	32	*O.latibracteata* Jurtz.	Helan Mountain	38°39'46.59"N, 105°49'20.25"E	X. Zhao 1951	2019
	33	*O.qilianshanica* C.W.Chang & C.L.Zhang	Jinqiang River	Unknown	J.Q. Wang 710113	Unknown
Section Lycotriche	34	*O.aciphylla* Ledeb.	Jiji Spring Nature Reserve	38°59'43"N, 101°55'39"E	X. Zhao 1924	2019
Section Leucopodia	35	*O.squammulosa* Candolle	Shaochagou	35°42'57.20"N, 105°2'21.20"E	X. Zhao 1928	2019

Seed shapes and surface sculpturing were classified according to previous studies on the microscopic morphology of Fabaceae seeds (Bojňanský and Fargašová 2007; Vural et al. 2008; Al-Ghamdi 2011; Meyers et al. 2013; Erkul et al. 2015). Based on previous studies and observations of seed morphology in the genus *Oxytropis*, seven seed traits, including four quantitative and three qualitative traits, were selected for morphometric analysis in the present study (Erkul et al. 2015). The selected characteristics and their states for cluster analysis were as follows: 1. seed length (mm); 2. seed width (mm); 3. seed length/width ratio; 4. seed shape: cardiform (0), prolonged (1), reniform (2), quadratic (3), prolonged semi-elliptic (4); 5. seed surface sculpturing: scaled (0), rugulate (1), lophate with stellated testa cells (2), simple reticulate (3), rough (4), compound reticulate (5), lophate with rounded testa cells (6); 6. hilum position: central (0), terminal (1); 7. seed weight (g). For the seeds collected in the field, 300 mature and full seeds were randomly selected and their 100-seed weight was determined. For the seeds collected from a few specimens, we randomly selected 30 seeds and weighed the 10-seeds. The 100-seed weights determined from the seeds of the specimens in the cluster analysis were expressed as 10-seed weights multiplied by 10.

## ﻿Numerical analysis

Cluster analysis and principal component analysis (PCA) were performed using the Origin 2022 software (OriginLab Corporation 2022). The raw data matrix included quantitative traits, such as length, width, L/W ratio and weight, and qualitative characteristics, such as shape, sculpturing, and hilum position. The qualitative characteristics were coded using a presence/absence (0/1) matrix. Ward’s method was used for cluster analysis using Euclidean distance to interpret the morphological similarities among species. In the cluster analysis, Euclidean distance is one of the most commonly used distance measurements in hierarchical clustering, which can reflect the absolute differences of individual numerical characteristics, and were applied to analyze differences in the numerical size of dimensions (Raymond and Sylvia 1993; Farhana and Safwana 2018). The Ward error sum of squares method applies the concept of ANOVA to classification, resulting in richer clustering information that is rarely affected by abnormal data (Ward 1963; Szekely and Rizzo 2005). In the present study, to test the validity of the seed macro-and micromorphological traits, PCA was used to select taxonomically relevant qualitative and quantitative characteristics. It is usually used to distinguish between species within a given genus.

## ﻿Results

### ﻿Seed morphology

**Figure 1.**
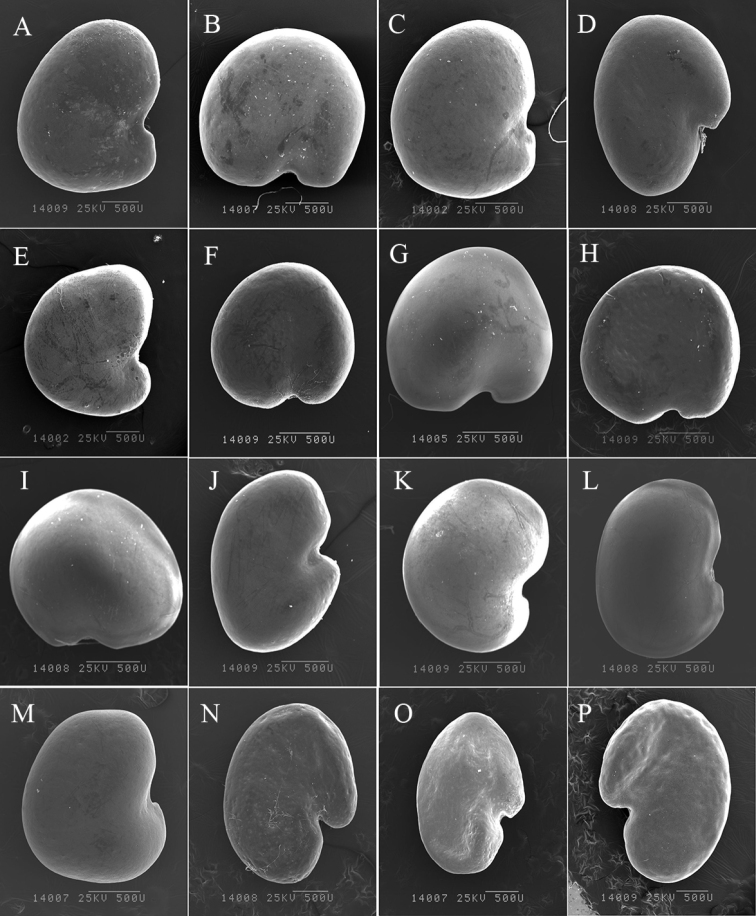
Seed shape of the studied species **A***O.ciliata***B***O.muricata* (MX) **C***O.muricata* (TM) **D***O.falcata* (AWC) **E***O.falcata* (MQ) **F***O.ochrantha* (XL) **G***O.ochrantha* (NMP) **H***O.bicolor* (U) **I***O.bicolor* (TM) **J***O.racemosa***K***O.myriophylla* (EDG) **L***O.myriophylla* (AG) **M***O.myriophylla* (MX) **N***O.neimonggolica***O***O.imbricata* (LC) **P***O.imbricata* (TR). Scale bar: 500 μm.

**Figure 2.**
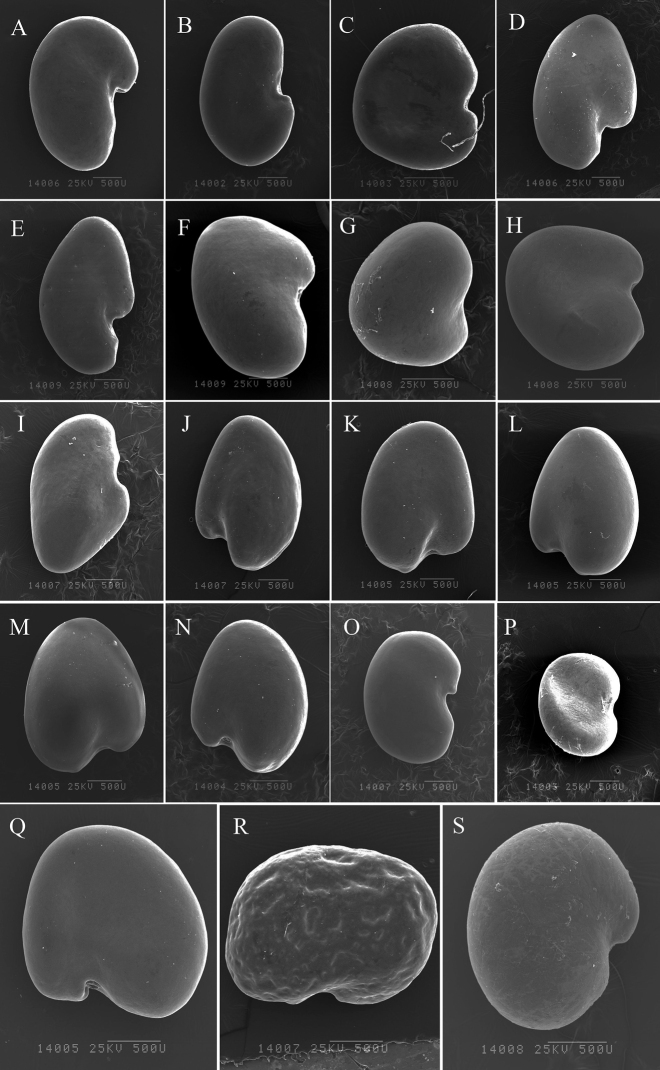
Seed shape of the studied species **A***O.coerulea* (TT) **B***O.coerulea* (EDG) **C***O.holanshanensis***D***O.xinglongshanica* (MX) **E***O.xinglongshanica* (XL) **F***O.glabra***G***O.kansuensis* (AZ) **H***O.kansuensis* (CT) **I***O.taochensis***J***O.ochrocephala* (NH) **K***O.ochrocephala* (XL) **L***O.ochrocephala* (MX) **M***O.ochrocephala* (JQ) **N***O.ochrocephala* (HG) **O***O.qinghaiensis***P***O.latibracteata***Q***O.qilianshanica***R***O.aciphylla***S***O.squammulosa*. Scale bar: 500 μm.

The studied seeds, all from the genus *Oxytropis*, had two main types of hilum positions, terminal and central, and five different types of seed shapes: prolonged semielliptic, reniform, prolonged reniform, quadratic, and cardiform (Table 2; Figs 1, 2). Hilum position was observed as terminal in *O.racemosa*, *O.neimonggolica*, *O.imbricata* (LC, TR), *O.coerulea* (TT, EDG), *O.xinglongshanica* (MX, XL), *O.glabra*, *O.taochensis*, and *O.ochrocephala* (NH, XL, MX, JQ, and HG). Hilum position was observed as central in *O.ciliata*, *O.muricata* (MX, TM), *O.falcata* (AWC, MQ), *O.ochrantha* (XL, NMP), *O.bicolor* (U, TM), *O.myriophylla* (EDG, AG, and MX), *O.holanshanensis*, *O.kansuensis* (AZ, CT), *O.qinghaiensis*, *O.latibracteata*, *O.qilianshanica*, *O.aciphylla*, and *O.squammulosa*. In addition, seed shapes could be separated into five groups (Table 2): a cardiform seed was found in *O.ciliata*, *O.muricata* (MX, TM), *O.falcata* (AWC, MQ), *O.ochrantha* (XL, NMP), *O.bicolor* (U, TM), *O.holanshanensis*, *O.kansuensis* (AZ, CT), and *O.squammulosa* (Table 2; Figs 1, 2); a prolonged reniform seed was observed in *O.racemosa*, *O.neimonggolica*, *O.imbricata* (LC, TR), *O.coerulea* (TT, EDG), *O.xinglongshanica* (MX, XL), *O.glabra*, and *O.taochensis* (Table 2; Figs 1, 2); a reniform seed was found in *O.myriophylla* (EDG, AG, MX), *O.qinghaiensis*, *O.latibracteata*, and *O.qilianshanica*; a quadratic seed was only found in *O.ochrocephala* (NH, XL, MX, JQ, HG); and finally, a prolonged semielliptic seed was only found in *O.aciphylla* (Table 2; Fig. 2).

**Table 2.** Seed morphological features of *Oxytropis* under scanning electron microscopy.

Section	Code	Species	Shape of seed	Sculpturing	Hilum position
Section Xerobia	1	*O.ciliata*	Cardiform	Scaled	Central
Section Polyadena	2	*O.muricata* (MX)	Cardiform	Rugulate	Central
	3	*O.muricata* (TM)	Cardiform	Rugulate	Central
Section Falcicarpae	4	*O.falcata* (AWC)	Cardiform	Rugulate	Central
	5	*O.falcata* (MQ)	Cardiform	Rugulate	Central
Section Baicalia	6	*O.ochrantha* (XL)	Cardiform	Lophate with stellated testa cells	Central
	7	*O.ochrantha* (NMP)	Cardiform	Rugulate	Central
	8	*O.bicolor* (U)	Cardiform	Rough	Central
	9	*O.bicolor* (TM)	Cardiform	Simple reticulate	Central
	10	*O.racemosa*	Prolonged Reniform	Rough	Terminal
	11	*O.myriophylla* (EDG)	Reniform	Rugulate	Central
	12	*O.myriophylla* (AG)	Reniform	Rough	Central
	13	*O.myriophylla* (MX)	Reniform	Rugulate	Central
Section Neimonggolicae	14	*O.neimonggolica*	Prolonged Reniform	Scaled	Terminal
Section Eumorpha	15	*O.imbricata* (LC)	Prolonged Reniform	Rugulate	Terminal
	16	*O.imbricata* (TR)	Prolonged Reniform	Rough	Terminal
	17	*O.coerulea* (TT)	Prolonged Reniform	Rugulate	Terminal
	18	*O.coerulea* (EDG)	Prolonged Reniform	Rugulate	Terminal
	19	*O.holanshanensis*	Cardiform	Compound reticulate	Central
Section Mesogaea	20	*O.xinglongshanica* (MX)	Prolonged Reniform	Lophate with stellated testa cells	Terminal
	21	*O.xinglongshanica* (XL)	Prolonged Reniform	Lophate with stellated testa cells	Terminal
	22	*O.glabra*	Prolonged Reniform	Rugulate	Terminal
	23	*O.kansuensis* (AZ)	Cardiform	Rugulate	Central
	24	*O.kansuensis* (CT)	Cardiform	Rugulate	Central
	25	*O.taochensis*	Prolonged Reniform	Lophate with stellated testa cells	Terminal
	26	*O.ochrocephala* (NH)	Quadratic	Rugulate	Terminal
	27	*O.ochrocephala* (XL)	Quadratic	Rugulate	Terminal
	28	*O.ochrocephala* (MX)	Quadratic	Rugulate	Terminal
	29	*O.ochrocephala* (JQ)	Quadratic	Rugulate	Terminal
	30	*O.ochrocephala* (HG)	Quadratic	Rugulate	Terminal
	31	*O.qinghaiensis*	Reniform	Compound reticulate	Central
Section Oxytropis	32	*O.latibracteata*	Reniform	Rugulate	Central
	33	*O.qilianshanica*	Reniform	Rough	Central
Section Lycotriche	34	*O.aciphylla*	Prolonged Semielliptic	Simple reticulate	Central
Section Leucopodia	35	*O.squammulosa*	Cardiform	Lophate with rounded testa cells	Central

The seeds ranged from 1.27 mm (*O.kansuensis* (AZ)) to 2.57 mm (*O.coerulea* (EDG)) in length and from 1.18 mm (*O.qinghaiensis*) to 2.02 mm (*O.holanshanensis*) in width (Table 3). The lowest length/width ratio (0.89) was observed in *O.ochrocephala* (JQ), while the highest (1.55) was found in *O.imbricata* (LC). The lightest seeds were measured in *O.qinghaiensis* at 0.1058 g, while the heaviest seeds were measured in *O.ciliata* at 0.3521 g (Table 3).

**Table 3.** Seed morphological features of *Oxytropis* under stereoscopic microscopy.

Section	Code	Species	Length Min. (mean ± SD) max./mm	Width Min. (mean ± SD) max./mm	L/W ratio	Seed weight/g
Section Xerobia	1	*O.ciliata*	1.54(2.43±0.36)3.28	1.34(2.05±0.28)2.8	1.19±0.07	0.3521±0.0236
ection *Polyadena*	2	*O.muricata* (MX)	1.38(2.02±0.3)2.91	1.1(1.78±0.26)2.38	1.14±0.07	0.2627±0.0041
	3	*O.muricata* (TM)	1.41(2.04±0.29)2.66	1.15(1.66±0.23)2.03	1.24±0.18	0.248±0.013
Section Falcicarpae	4	*O.falcata* (AWC)	1.59(2.14±0.25)2.79	1.42(1.87±0.18)2.35	1.15±0.13	0.2981± 0.0106
	5	*O.falcata* (MQ)	1.7(2.09±0.14)2.5	1.45(1.86±0.17)2.15	1.13±0.13	0.323±0.005
Section Baicalia	6	*O.ochrantha* (XL)	1.29(1.65±0.17)2.09	1.18(1.53±0.16)2	1.07±0.07	0.2148±0.0091
	7	*O.ochrantha* (NMP)	1.16(1.49±0.17)1.84	1.15(1.35±0.12)1.6	1.1±0.07	0.1732±0.0021
	8	*O.bicolor* (U)	1.09(1.57±0.23)1.98	1.21(1.49±0.17)1.88	1.05±0.06	0.146±0.013
	9	*O.bicolor* (TM)	1.32(1.74±0.21)2.31	1.23(1.65±0.23)2.32	1.06±0.08	0.1326±0.0086
	10	*O.racemosa*	1.24(1.71±0.17)2.19	0.77(1.23±0.14)1.5	1.4±0.12	0.1668±0.0128
	11	*O.myriophylla* (EDG)	1.05(1.56±0.21)2.14	0.81(1.25±0.19)1.64	1.26±0.1	0.1290±0.0004
	12	*O.myriophylla* (AG)	1.26(1.59±0.13)1.93	0.94(1.32±0.16)1.63	1.22±0.09	0.1231±0.0007
	13	*O.myriophylla* (MX)	1.06(1.67±0.23)2.1	1.04(1.45±0.16)1.9	1.15±0.07	0.1349±0.0043
Section Neimonggolicae	14	*O.neimonggolica*	1.85(2.11±0.12)2.32	1.54(1.72±0.12)1.98	1.23±0.04	0.326±0.01
Sectio*n Eumorpha*	15	*O.imbricata* (LC)	1.77(2.44±0.27)2.93	1.1(1.59±0.2)2.05	1.54±0.11	0.3188±0.0054
	16	*O.imbricata* (TR)	1.79(2.36±0.31)3.13	1.08(1.56±0.27)2.34	1.52±0.12	0.3264±0.0112
	17	*O.coerulea* (TT)	1.66(2.39±0.25)2.96	1.22(1.69±0.21)2.11	1.43±0.12	0.2799±0.0016
	18	*O.coerulea* (EDG)	1.99(2.57±0.16)2.92	1.44(1.86±0.15)2.2	1.39±0.09	0.2986± 0.0013
	19	*O.holanshanensis*	1.81(2.21±0.19)2.66	1.43(2.02±0.22)2.58	1.1±0.09	0.3264±0.0062
Section Mesogaea	20	*O.xinglongshanica* (MX)	1.56(2.32±0.32)3.22	1.29(1.93±0.29)2.69	1.21±0.1	0.2914±0.0038
	21	*O.xinglongshanica* (XL)	1.47(2.23±0.23)2.7	1.42(1.77±0.16)2.24	1.26±0.11	0.2763±0.0103
	22	*O.glabra*	0.93(1.78±0.33)2.63	0.84(1.53±0.27)2.23	1.16±0.09	0.1892±0.0066
	23	*O.kansuensis* (AZ)	0.87(1.27±0.2)1.9	0.91(1.28±0.17)1.69	0.99±0.09	0.1074±0.0057
	24	*O.kansuensis* (CT)	0.95(1.38±0.13)1.65	1.05(1.44±0.17)1.77	0.97±0.11	0.1260±0.0044
	25	*O.taochensis*	1.54(2.09±0.25)2.73	1.08(1.55±0.18)1.89	1.36±0.11	0.2236±0.0134
	26	*O.ochrocephala* (NH)	1.22(1.73±0.22)2.23	1.35(1.91±0.23)2.53	0.9±0.06	0.2719±0.0043
	27	*O.ochrocephala* (XL)	1.1(1.63±0.17)2.03	1.23(1.77±0.24)2.39	0.92±0.07	0.2517±0.0103
	28	*O.ochrocephala* (MX)	1.28(1.64±0.17)2.01	1.56(1.82±0.12)2.14	0.9±0.07	0.2417±0.0065
	29	*O.ochrocephala* (JQ)	0.92(1.56±0.23)2.06	1.23(1.75±0.21)2.43	0.89±0.08	0.2506±0.0098
	30	*O.ochrocephala* (HG)	1.02(1.66±0.26)2.28	1.43(1.8±0.14)2.27	0.92±0.11	0.2854±0.0123
	31	*O.qinghaiensis*	1.2(1.56±0.18)1.99	0.93(1.18±0.11)1.56	1.33±0.1	0.1058±0.0087
Section Oxytropis	32	*O.latibracteata*	1.5(2.05±0.25)2.64	1.2(1.69±0.21)2.19	1.22±0.1	0.2368±0.0106
	33	*O.qilianshanica*	1.38(1.57±0.08)1.71	1.09(1.31±0.1)1.49	1.2±0.05	0.112±0.008
Section Lycotriche	34	*O.aciphylla*	1.36(1.99±0.28)2.81	1.01(1.43±0.21)1.98	1.39±0.12	0.1822±0.0094
Section Leucopodia	35	*O.squammulosa*	1.22(1.81±0.25)2.61	0.95(1.62±0.29)2.37	1.13±0.09	0.2070±0.0117

### ﻿Surface sculpturing

**Figure 3.**
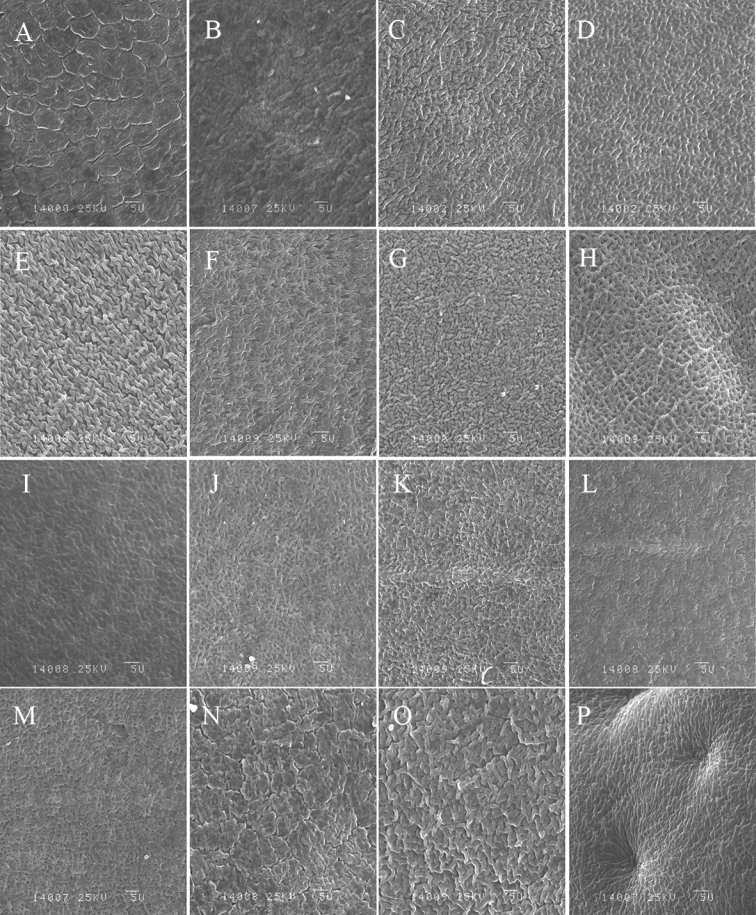
Seed surface sculpturing of the studied species **A***O.ciliata***B***O.muricata* (MX) **C***O.muricata* (TM) **D***O.falcata* (AWC) **E***O.falcata* (MQ) **F***O.ochrantha* (XL) **G***O.ochrantha* (NMP) **H***O.bicolor* (U) **I***O.bicolor* (TM) **J***O.racemosa***K***O.myriophylla* (EDG) **L***O.myriophylla* (AG) **M***O.myriophylla* (MX) **N***O.neimonggolica***O***O.imbricata* (LC) **P***O.imbricata* (TR). Scale bar: 5 μm.

**Figure 4.**
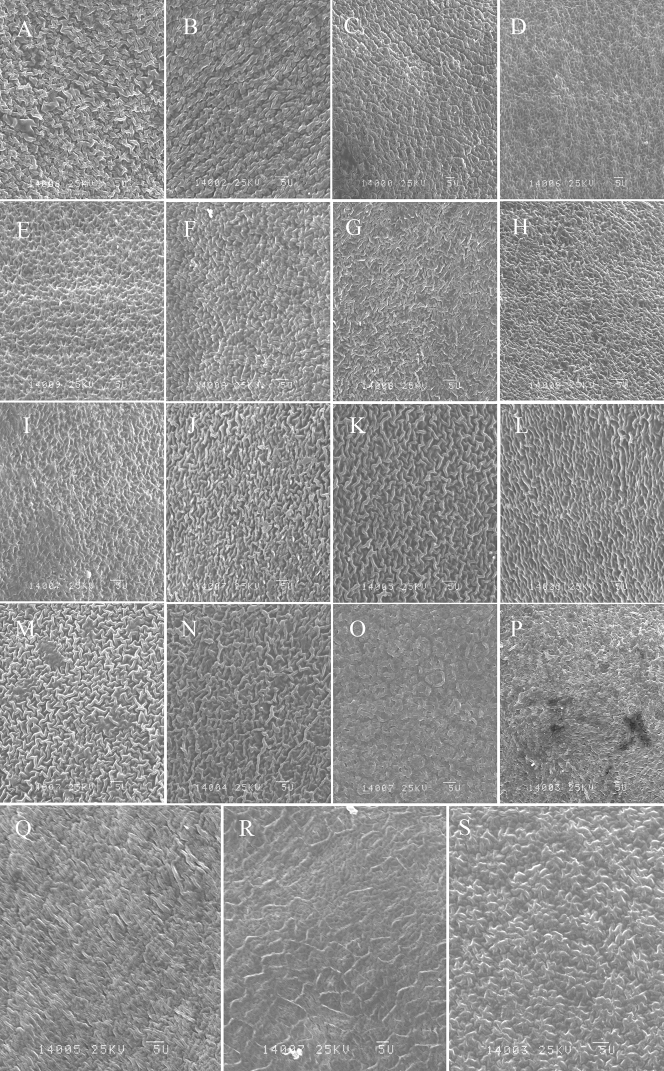
Seed surface sculpturing of the studied species **A***O.coerulea* (TT) **B***O.coerulea* (EDG) **C***O.holanshanensis***D***O.xinglongshanica* (MX) **E***O.xinglongshanica* (XL) **F***O.glabra***G***O.kansuensis* (AZ) **H***O.kansuensis* (CT) **I***O.taochensis***J***O.ochrocephala* (NH) **K***O.ochrocephala* (XL) **L***O.ochrocephala* (MX) **M***O.ochrocephala* (JQ) **N***O.ochrocephala* (HG) **O***O.qinghaiensis***P***O.latibracteata***Q***O.qilianshanica***R***O.aciphylla***S***O.squammulosa*. Scale bar: 5 μm.

Seven different seed surface sculpturing patterns were observed: scaled, regulate, lophate with stellated testa cells, simple reticulate, rough, compound reticulate, and lophate with rounded testa cells (Table 2; Figs 3, 4). The regulate sculpturing pattern was common in most taxa and was predominant in *O.muricata* (MX, TM), *O.falcata* (AWC, MQ), *O.ochrantha* (NMP), *O.myriophylla* (EDG, MX), *O.kansuensis* (AZ, CT), *O.latibracteata*, *O.imbricata* (LC), *O.coerulea* (TT, EDG), *O.glabra*, and *O.ochrocephala* (NH, XL, MX, JQ, and HG) (Table 2; Figs 3, 4). The simple reticulate sculpturing pattern was predominant in *O.bicolor* (U) and *O.aciphylla*, while the compound reticulate sculpturing pattern was predominant in *O.holanshanensis* and *O.qinghaiensis* (Table 2; Figs 3, 4). The scaled sculpturing pattern was predominant in *O.ciliata* and *O.neimonggolica*, while the rough sculpturing pattern was predominant in *O.bicolor*, *O.myriophylla* (AG), *O.qilianshanica*, *O.racemosa*, and *O.imbricata* (TR) (Table 2; Figs 3, 4). Lastly, the lophate pattern with stellated testa cells was predominant in *O.ochrantha* (XL), *O.xinglongshanica* (MX, XL), and *O.taochensis*, while the lophate pattern with rounded testa cells was only found in *O.squammulosa* (Table 2; Figs 3, 4).

### ﻿Numerical analysis

**Figure 5.**
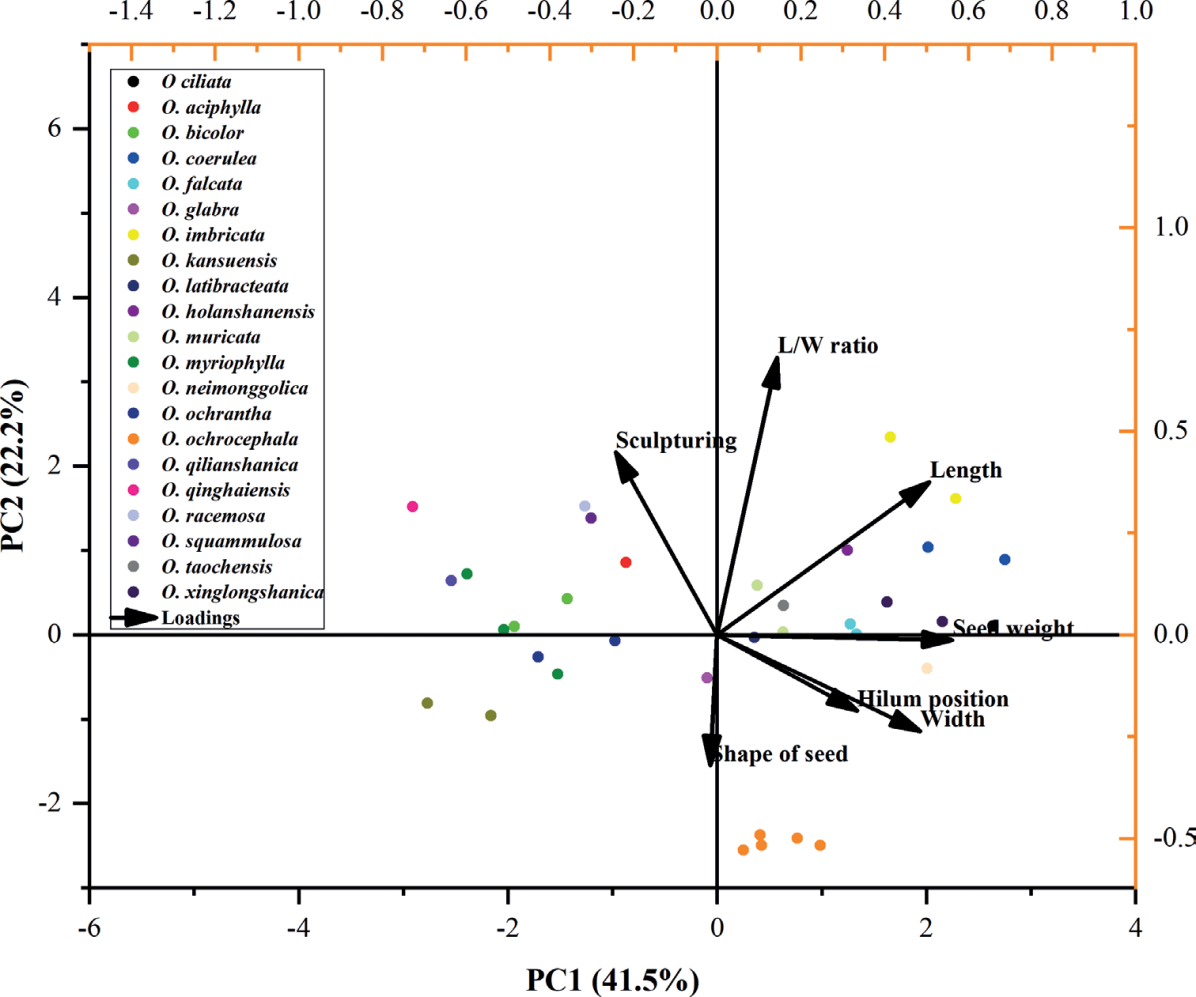
PCA for 35 samples belonging to 21 *Oxytropis* species based on seed morphological characters. Dots of different colors represent different species, and dots of the same color represent different populations of the same species.

**Figure 6.**
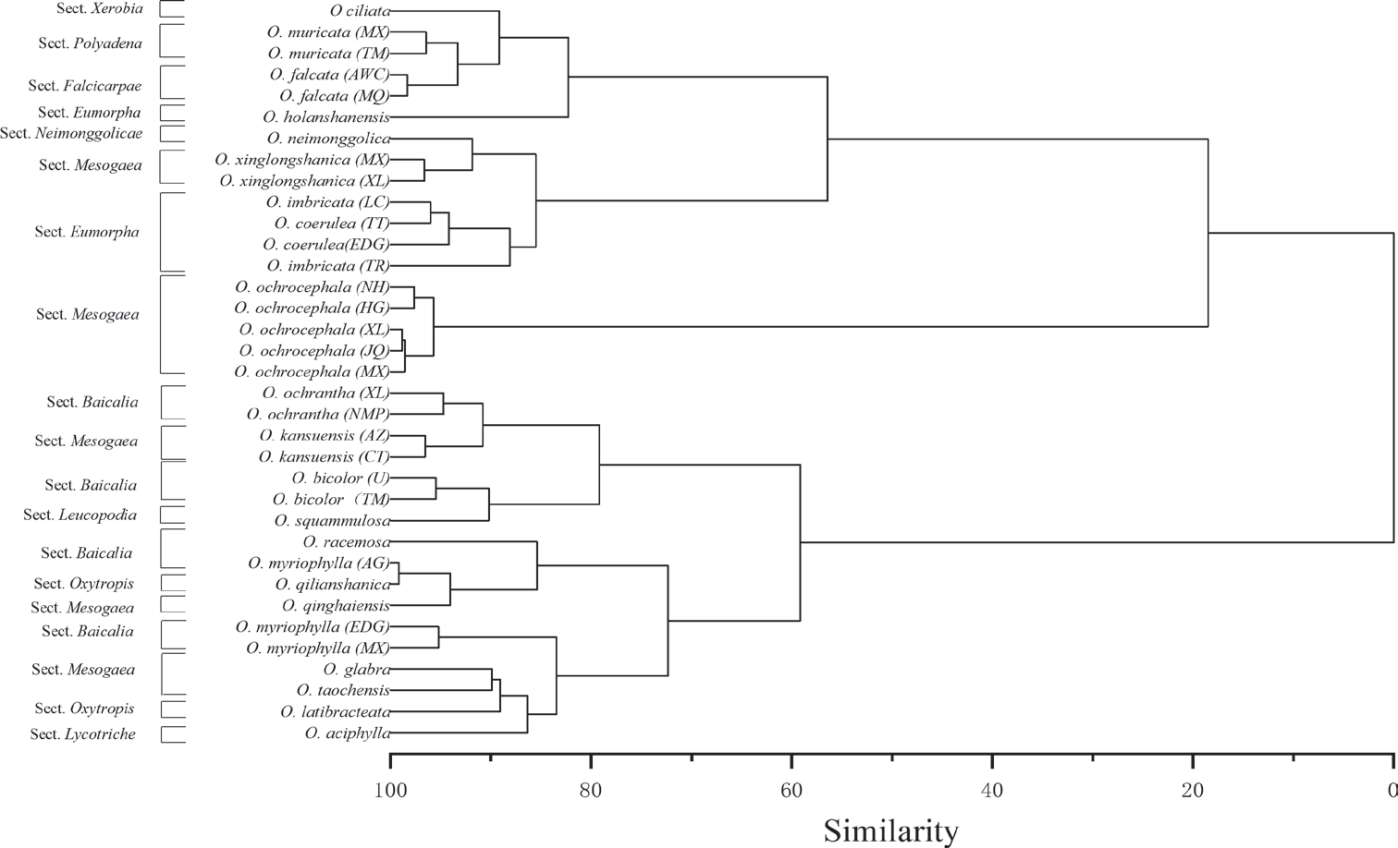
Phenogram for 35 samples belonging to 21 *Oxytropis* species based on based on seed morphological characters.

In the present study, principal components analysis (PCA) indicates three groups of traits, which explain 82.81% of the total variation (Table 4). The first principal component (PC1) exhibited 41.51% of the variability, which had a high loading component of the seed length, width, and weight. The second PC (PC2) accounted for 22.18% of the variation and was strongly associated with L/W ratio and sculpturing, whereas the third PC (PC3) contained 19.12% of the variability in which hilum position and seed shape were important. As shown in Fig. 5, the scatter points for the same species are closely aggregated, such as the five samples of *O.ochrocephala* (NH, XL, MX, JQ, and HG), indicating that samples from different populations within the same species had similar characteristics. However, the arrangement of 21 species belonging to 10 sections does not show a certain regularity. For example, species belonging to different sections are also arranged together, indicating that the seed morphological characteristics of *Oxytropis* species does not have regularity within the section. Cluster analysis reflects the similarity among species based on the anatomical characteristics and delimitation of these groups. Our phenograms of the quantitative and qualitative data showed three primary clusters (Fig. 6). The first cluster included *O.ciliata*, *O.muricata* (MX, TM), *O.falcata* (AWC, MQ), *O.holanshanensis*, *O.neimonggolica*, *O.xinglongshanica* (MX, XL), *O.imbricata* (LC), *O.coerulea* (TT, EDG), and *O.imbricata* (TR). The second cluster only contained *O.ochrocephala* (NH, XL, MX, JQ, and HG). The third cluster included *O.ochrantha* (XL, NMP), *O.kansuensis* (AZ, CT), *O.bicolor* (U, TM), *O.squammulosa*, *O.racemosa*, *O.myriophylla* (AG), *O.qilianshanica*, *O.qinghaiensis*, *O.myriophylla* (EDG, MX), *O.glabra*, *O.taochensis*, *O.latibracteata*, and *O.aciphylla*.

**Table 4.** PCA variable loading characters of seed micro-morphology of studied *Oxytropis* species.

PCA variable loadings	PC1	PC2	PC3
Shape of seed	-0.01682	-0.32175	0.7042
Sculpturing	-0.24237	0.44835	0.07685
Hilum position	0.33478	-0.18679	0.5441
Length	0.50744	0.37518	0.00087
Width	0.48498	-0.23663	-0.28718
L/W ratio	0.14335	0.68091	0.32905
Seed weight	0.56183	-0.01324	-0.10682
Eigenvalue	2.90597	1.55275	1.33831
Variability/%	41.51384	22.18208	19.11869
Cumulative/%	41.51384	63.69592	82.81461

## ﻿Discussion

Seed morphology of the investigated species was determined for the first time in the present study. Seed characteristics, such as coat pattern, shape, and size, have been shown to be important for the classification within genera of Fabaceae species (Lersten and Gunn 1981; Solum and Lockerman 1991; López et al. 2000; Al-Gohary and Mohamed 2007; Salimpour et al. 2007; Vural et al. 2008; Venora et al. 2009; Zorić et al. 2010; Celep et al. 2012; De-Paula and Oliveira 2012; Kaya and Dirmenci 2012; Lantieri et al. 2013). Previous studies have shown that seed shape and hilum position are taxonomically significant and can therefore be used for the classification of taxa at the genus or even species level (López et al. 2000; Salimpour et al. 2007; Vural et al. 2008). The five main types of seed shapes observed in the present study were consistent with previous findings on *Oxytropis* (Erkul et al 2015). The seed shapes of different populations of the same *Oxytropis* species were highly consistent, indicating that they were relatively constant within species. Particularly, *O.ochrocephala* and *O.kansuensis* are easily confused, as they are morphologically difficult to distinguish and are both abundant in the northwest China (Zhu et al. 2010). However, our observations demonstrate that these two species can be distinguished based on their seed shape; *O.ochrocephala* has a quadratic seed, whereas *O.kansuensis* has a cardiform seed. These results indicate that seed shape might be a useful taxonomic marker for some *Oxytropis* species. However, similar seed shapes exist in other species of the genus *Oxytropis* and other groups of Fabaceae (Erkul et al. 2015). Thus, they should be considered in combination with other macro-morphological characteristics when applied to species identification within the genus *Oxytropis*.

The sculpturing pattern of seeds is thought to provide useful information for the infrageneric classification of some genera of Fabaceae (Salimpour et al. 2007; Vural et al. 2008; Kamala and Aydin 2018; Rashid et al. 2020). Farrington et al. (2008) proposed that *Oxytropis* seed coat micromorphology and anatomy can be used to distinguish *Oxytropis* from its sister taxon, *Astragalus*. However, studies have shown that the taxonomic value of seed sculpturing patterns in *Astragalus* and *Oxytropis* species is limited. For example, a study that examined 48 species of Turkish *Astragalus* found only two distinct seed coat morphological types (rugulate and rugulate-reticulate) (Vural et al. 2008). Similarly, Shemetova et al. (2018) recognised two main types of seed surface in the genus *Astragalus*: reticulate and indistinct primary sculpture. However, these seed sculpturing patterns have also been observed in the genus *Oxytropis*. Farrington et al. (2008) found that Alaskan *Oxytropis* (15 taxa) has rugulate, rugulate-reticulate, and lophate sculpturing patterns. Consistently, Erkul et al. (2015) reported three types of seed sculpturing patterns in *Oxytropis*, namely rugulate, rugulate-reticulate, and lophate, and proposed that seed characteristics are not useful for separating the genera *Oxytropis* and *Astragalus*. Furthermore, Meyers et al. (2013) proposed that seed coat types among the Alaskan members of *Oxytropis* are highly variable at the species level and cannot be used for species identification. Our results supported this hypothesis because seed sculpturing patterns are variable within some species, including *O.ochrantha* (XL, NMP), *O.bicolor* (U, TM), *O.myriophylla* (EDG, AG, and MX), and *O.imbricata* (LC, TR), suggesting that seed sculpturing pattern has a limited taxonomic value. Interestingly, in the present study, the seed sculpturing pattern appeared to be conserved differently within different sections. Seed coat patterns were stable within some species in the Section Mesogaea, such as *O.ochrocephala*, *O.kansuensis*, and *O.xinglongshanica*, but highly variable in the species of the sections *Baicalia* and *Eumorpha*. Therefore, the taxonomic significance of seed sculpturing pattern should be comprehensively analysed using a broader sample.

Previous studies on *Oxytropis* have suggested that seed characteristics, such as size (length, width, and length/width ratio), shape, surface sculpturing, and weight have low taxonomic value at the infrageneric level (Solum and Lockerman 1991; Bojňanský and Fargašová 2007; Farrington et al. 2008; Meyers et al. 2013; Erkul et al. 2015). However, most of these studies only subjectively compared their quantitative traits without a systematic analysis such as a cluster analysis. Only Erkul et al. (2015) systematically analysed the seed traits in 13 *Oxytropis* species from Turkey, but they did not explore the variation in seed traits at the species level because of sampling limitations. In the present study, the results of the cluster analysis showed that, except for *O.myriophylla*, different populations of the same species were clustered into one clade, indicating that the seed traits of *Oxytropis* are useful for the identification of taxa at the species level. However, species belonging to different sections were present in the same clade, indicating that seed characteristics have low taxonomic value at the section level. The results of the PCA also supported the former view that populations within the same species cluster together, while the distribution of samples of different species does not show a certain regularity. Furthermore, the first PC of the PCA provided a highly dominant variability of 41.51%, the characteristics with major scores that contributed to the formation of the groups were quantitative characteristics, such as length, width, and weight of seed. The second and third PCs are mainly qualitative characteristics, accounting for 41.3% of the total variance. These results suggest that even though quantitative traits and some qualitative traits, such as seed sculpturing patterns, are highly variable within species, these traits still play an important role in systematic analysis. Therefore, it is necessary to comprehensively analyse qualitative and quantitative characteristics in future research into *Oxytropis* seed morphology.

To date, a comprehensive phylogenetic study of the genus *Oxytropis* has not been carried out. Moreover, even though several studies have utilized DNA barcodes such as ITS, *trnL-F*, and *psbA-trnH* to investigate the molecular phylogeny of *Oxytropis* in northwest China, the low genetic difference of these barcodes among species makes it difficult to distinguish species within this genus and solve the phylogenetic relationship among its species (Li et al. 2011; Gao et al. 2013; Lu et al. 2014). Therefore, the phylogenetic reliability of seed traits in *Oxytropis* cannot be confirmed. More detailed molecular phylogenetic studies and more extensive taxon sampling are needed to discover the correlation between seed features and genus taxonomy.

## ﻿Conclusions

Our results suggest that the seed traits of *Oxytropis* are helpful for identifying taxa at the species level, but have low taxonomic value at the section level. Seed shape was constant within species and was useful for species delimitation in the genus *Oxytropis* when combined with other macroscopic traits. The seed sculpturing patterns were highly variable at the species level and could not be used for species identification. Although quantitative traits and some qualitative traits, such as seed sculpturing patterns, are highly variable within species, these traits still play an important role in PCA and cluster analysis. The results of the PCA and cluster analysis showed that different populations of the same species were clustered into one clade, indicating that in *Oxytropis*, seed traits are useful for the identification of taxa at the species level. However, species belonging to different sections also clustered into the same clade, indicating that seed characteristics have low taxonomic value at the section level.
